# Sintokamide A Is a Novel Antagonist of Androgen Receptor That Uniquely Binds Activation Function-1 in Its Amino-terminal Domain*

**DOI:** 10.1074/jbc.M116.734475

**Published:** 2016-08-30

**Authors:** Carmen A. Banuelos, Iran Tavakoli, Amy H. Tien, Daniel P. Caley, Nasrin R. Mawji, Zhenzhen Li, Jun Wang, Yu Chi Yang, Yusuke Imamura, Luping Yan, Jian Guo Wen, Raymond J. Andersen, Marianne D. Sadar

**Affiliations:** From the ‡ Department of Genome Sciences Centre, British Columbia Cancer Agency, 675 West 10th Avenue, Vancouver, British Columbia V5Z 1L3, Canada,; § Institute of Clinical Medicine, First Affiliated Hospital of Zhengzhou University, Zhengzhou, Henan 450052, China,; ¶ Chemistry and Earth and Ocean Sciences, University of British Columbia, 2036 Main Mall, Vancouver, British Columbia V6T 1Z1, Canada, and; ‖ Urodynamic Center, First Affiliated Hospital of Zhengzhou, Zhengzhou University Hospital, Zhengzhou, Henan 450052, China

**Keywords:** androgen receptor, drug development, intrinsically disordered protein, prostate cancer, steroid hormone receptor, transcription factor

## Abstract

Androgen receptor (AR) is a validated drug target for all stages of prostate cancer including metastatic castration-resistant prostate cancer (CRPC). All current hormone therapies for CRPC target the C-terminal ligand-binding domain of AR and ultimately all fail with resumed AR transcriptional activity. Within the AR N-terminal domain (NTD) is activation function-1 (AF-1) that is essential for AR transcriptional activity. Inhibitors of AR AF-1 would potentially block most AR mechanisms of resistance including constitutively active AR splice variants that lack the ligand-binding domain. Here we provide evidence that sintokamide A (SINT1) binds AR AF-1 region to specifically inhibit transactivation of AR NTD. Consistent with SINT1 targeting AR AF-1, it attenuated transcriptional activities of both full-length AR and constitutively active AR splice variants, which correlated with inhibition of growth of enzalutamide-resistant prostate cancer cells expressing AR splice variants. *In vivo*, SINT1 caused regression of CRPC xenografts and reduced expression of prostate-specific antigen, a gene transcriptionally regulated by AR. Inhibition of AR activity by SINT1 was additive to EPI-002, a known AR AF-1 inhibitor that is in clinical trials (NCT02606123). This implies that SINT1 binds to a site on AF-1 that is unique from EPI. Consistent with this suggestion, these two compounds showed differences in blocking AR interaction with STAT3. This work provides evidence that the intrinsically disordered NTD of AR is druggable and that SINT1 analogs may provide a novel scaffold for drug development for the treatment of prostate cancer or other diseases of the AR axis.

## Introduction

Approximately 20–40% of patients treated for localized prostate cancer will have recurrence. The current therapy for these patients is hormone therapy such as orchiectomy or luteinizing hormone-releasing hormone analogs followed by antiandrogens and 17α-hydroxylase/17,20-lyase (CYP17) inhibitors. Current hormone therapies all target the ligand-binding domain (LBD)^2^ of androgen receptor (AR) and will eventually fail, giving rise to lethal metastatic castration-resistant prostate cancer (mCRPC). CRPC is characterized by a recurrent rise of serum prostate-specific antigen (PSA) levels despite castrate blood levels of androgen. *PSA* is an androgen-regulated gene that is dependent upon AR transactivation. Therefore, a rising PSA level despite castrate serum levels of androgen suggests continued AR transactivation. One probable AR mechanism of resistance to hormone therapies associated with increasing PSA levels is expression of constitutively active AR splice variants that lack the LBD.

Transcriptional activity of AR resides within the activation function-1 (AF-1) region, which is essential for transcriptional activities of both full-length AR (FL-AR) and constitutively active AR splice variants lacking the LBD (1–3). AF-1 comprises two subregions: transcriptional activation unit 1 (Tau1) and Tau5. Tau1 resides between residues 101 and 370, and Tau5 resides between residues 360 and 485 (3). The search for small molecules that directly interact with AR AF-1 has yielded one class of molecules to date, EPI-001, its stereoisomers including EPI-002 (4, 5), and imaging agent ^123^I-EPI-002 (6). The prodrug of EPI-002, EPI-506, is currently in Phase 1/2 clinical trials for prostate cancer patients in the United States and Canada (NCT02606123).

Sintokamide A (SINT1) is a natural compound isolated and purified from the marine sponge *Dysidea* sp. (7). Interest in SINT1 is drawn from the fact that it blocks transactivation of the AR NTD and inhibits AR-dependent proliferation of prostate cancer cells *in vitro* (7). Here the specificity of SINT1 toward AR and its ability to inhibit the growth of CRPC xenografts were investigated. The mechanism of action of SINT1 involved binding to AF-1 to specifically block the transcriptional activities of FL-AR and splice variant ARs without attenuating the transcriptional activities of structurally related steroid hormone receptors. SINT1 blocked transactivation of AR NTD induced by stimulation of the PKA pathway, but contrary to EPI, SINT1 had no effect on IL-6-induced transactivation of AR NTD. This suggests that SINT1 binds to a different region of AF-1 compared with EPI. Consistent with SINT1 binding to a unique site on AF-1 from EPI, SINT1 did not prevent interaction between endogenous AR and STAT3 in response to IL-6, whereas EPI did. Lastly, the additive affect observed when SINT1 was combined with EPI was consistent with EPI and SINT1 having different mechanisms of action. *In vivo*, SINT1 reduced proliferation and caused regression of CRPC xenografts as well as decreased expression of the AR-regulated gene *PSA*.

## Experimental Procedures

### 

#### 

##### Materials

Antibodies against AR (AR-N20, N terminus, rabbit polyclonal; sc-816, Santa Cruz Biotechnology), PSA (clone ER-PR8, mouse monoclonal; M0750, Dako) and anti-His (H-15; sc-803, Santa Cruz Biotechnology) were from the sources indicated. β-Actin antibody (AC-15, mouse monoclonal; ab6276) and anti-biotin (rabbit polyclonal; ab1227) were from Abcam. STAT3 (mouse monoclonal, 124H6) was from Cell Signaling Technology. Anti-streptavidin (IRDye® 680LT streptavidin, LIC-926-68030) was from LI-COR Biosciences. SINT1 is a natural compound (7), and LPY19, LPY30, LPY31, and EPI-053 were synthesized (5). EPI-002 was provided by NAEJA Pharmaceutical Inc. The synthetic androgen R1881 was purchased from PerkinElmer Life Sciences. Enzalutamide (MDV-3100) was purchased from Omega Chem. Bicalutamide was a gift from Dr. Marc Zarenda, AstraZeneca Pharmaceuticals. Interleukin-6 was purchased from R&D Systems (Minneapolis, MN). RPMI 1640 medium was purchased from Life Technologies. All other chemicals including progesterone (4-pregnene-3,20-dione) and dexamethasone were obtained from Sigma-Aldrich unless otherwise stated. PSA (6.1 kb)-luciferase, probasin (PB)-luciferase, PRE-luciferase, GRE-luciferase, 5xGal4UAS-TATA-luciferase, AR(1–558)-Gal4 DNA-binding domain (DBD), AR-YFP, and AR^var567es^ have been described (4, 8).

##### Cell Lines

LNCaP cells were maintained in RPMI 1640 medium supplemented with 5% fetal bovine serum (FBS). LNCaP cells were authenticated by short tandem repeat analysis and tested to ensure that they were mycoplasma-free by DDC Medical. LNCaP95 cells expressing FL-AR and AR-V7 were provided by Dr. Plymate (University of Washington). These cells were maintained in RPMI 1640 medium supplemented with 10% dextran-coated charcoal-stripped FBS.

##### Animals

NOD-SCID (6–8 week-old) male mice were maintained in the Animal Care Facility at the British Columbia Cancer Research Centre. All animal studies adhered to the relevant regulatory standards protocols approved by the University of British Columbia Animal Care Committee (Vancouver, British Columbia, Canada). Metacam (1 mg/kg, 0.05 ml/10 g of body weight) was injected subcutaneously prior to any surgery. Animals were anesthetized using isoflurane anesthesia and euthanized by CO_2_.

##### Steroid Receptor Specificity

LNCaP cells were transfected with the reporter plasmid PSA(6.1kb)-luciferase, PRE-luciferase, or GRE-luciferase and expression vector for progesterone receptor (PR)-β or glucocorticoid receptor (GR)-α in serum-free, phenol red-free medium using Lipofectin (Invitrogen). Sixteen hours later, the cells were pretreated with 10 μm SINT1, 10 μm bicalutamide, or vehicle for 1 h before addition of the corresponding ligand (1 nm R1881, 10 nm 4-pregnene-3,20-dione, or 10 nm dexamethasone). After 48 h of treatment, cells were lysed and analyzed for luciferase activities.

##### Binding Assay of SINT1 to Full-length AR

LNCaP cells were treated for 24 h with 20 μm modified SINT1 (LPY19, -30, and -31) or the vehicle dimethyl sulfoxide (DMSO). 25 μm EPI-053 (a modified EPI-001 analog) was used as positive control (5). Proteins were extracted with lysis buffer and subjected to click chemistry conditions for 3 h at 25 °C in buffer containing 0.1% SDS, 5% *t*-butyl alcohol, 100 μm tris[(1-benzyl-1*H*-1,2,3-triazol-4-yl)methyl]amine (Sigma), 1 mm tris(2-carboxyethyl)phosphine, 100 μm biotin-azide reagent, and 1 mm CuSO_4_. Samples were dialyzed in 50 mm HEPES, pH 8.0, 150 mm NaCl, 0.1% SDS, and 1% Triton X-100 to remove excess biotin-azide reagent. Biotinylated soluble receptors were captured with streptavidin-agarose resin. Protein-SINT1 complexes were separated by 10% SDS-PAGE and subjected to Western blotting analysis using AR-N20 (1:1000) and biotin (1:1000) antibodies. Proteins were visualized using ECL detection reagent (Amersham Biosciences).

##### Binding Assay of SINT1 to AR AF-1

Histidine-tagged AR AF-1 recombinant protein was expressed and purified as described (5). The binding reaction was carried out by mixing AR AF-1 protein with alkyne-containing LPY probes or DMSO at a 1:10 molar ratio and incubated in buffer containing 10 mm HEPES, pH 7.9, and 100 mm NaCl at 37 °C for 3 h. LPY probes were labeled with biotin by copper-catalyzed click chemistry reaction at 25 °C for 1 h (5). Samples were resolved by 10% SDS-PAGE, and Western blotting was performed to detect biotin and histidine-tagged AR AF-1 using IRDye® 680LT streptavidin (1:5000) and anti-His (1:1000) antibody, respectively.

##### Fluorescence Polarization

The Androgen Receptor Competitor Assay (Green) kit (Invitrogen) was used. AR LBD (25 nm) was incubated with 1 nm Fluormone^TM^ AL Green to achieve ∼80% saturation. The reactions with serial dilutions of test compounds were completed in 40-μl aliquots in triplicates. Displacement of Fluormone AL Green from AR LBD by the test compounds was measured by the change in fluorescence polarization using Infinite® M1000 (Tecan) with excitation at 470 nm and emission at 535 nm.

##### Transactivation of AR NTD

LNCaP cells were co-transfected with 5xGal4UAS-TATA-luciferase and AR-(1–558)-Gal4 DBD prior to pretreatment with 20 μm SINT1 or 25 μm EPI-002, which binds to the AR NTD to block AR transcriptional activity. NTD activity was induced by incubation with 50 μm forskolin or 50 ng/ml IL-6 for an additional 24 h (7, 8).

##### Co-immunoprecipitation

LNCaP cells were preincubated with EPI-002 (35 μm), SINT1 (20 μm), or DMSO (vehicle control) for 1 h before treatment for 6 h with 50 ng/ml IL-6. Whole cell lysates were prepared from harvested cells in modified radioimmune precipitation assay buffer. Cell lysates were precleared with 2 μg of normal mouse IgG and 20 μl of Protein A/G PLUS-agarose beads (Santa Cruz Biotechnology) to remove nonspecific bead binding. Total STAT3 was immunoprecipitated with 10 μg of antibody against STAT3 (124H6) or 10 μg of normal mouse IgG as a negative control followed by probing the immunoblot with anti-AR antibody (AR-N20; 1:1000) and anti-STAT3 antibody (124H6; 1:1000) and normalizing to levels of β-actin (1:10,000) as loading controls. The input lanes were loaded with 5 μg of the starting cell lysate. The relative density of AR for each immunoprecipitated sample was quantified by ImageJ software and normalized to levels of STAT3 pulled down.

##### SINT1 and EPI-002 Combination

LNCaP cells were transfected with AR-responsive PSA (6.1 kb)-luciferase, PB-luciferase, and ARR3-luciferase reporters. Forty-eight hours later, cells were pretreated for 1 h with SINT1 (0–24 μm), EPI-002 (0–31 μm), or a combination with a constant ratio of 1:1.3 for EPI-002/SINT1 concentration. Cells were then incubated with 1 nm R1881 for 48 h, and a luciferase assay was performed. IC_50_ values for SINT1 for the three reporters were calculated using GraphPad Prism (version 6.01; GraphPad Software). The combination index of SINT1 and EPI-002 was calculated using CalcuSyn software. Combination indices >1, 1, and <1 indicate antagonism, additive affect, and synergy, respectively.

##### Transcriptional Activity of AR Splice Variant

COS-1 cells (AR-negative) were co-transfected with the PB-luciferase reporter and the expression plasmid AR^var567es^ encoding the constitutively active AR splice variant that lacks the LBD. Six hours after transfection, cells were pretreated for 1 h with 20 μm SINT1, 25 μm EPI-002, 10 μm bicalutamide, 1 μm enzalutamide, or vehicle before incubation with R1881 for another 24 h. Luciferase activity was measured and normalized to protein concentration. Levels of expression of AR variant were determined by Western blotting using 10 μg of total protein extracted from same plates (3 wells per condition) using AR-N20 antibody (1:1000) and goat anti-rabbit HRP-conjugated secondary antibody (Santa Cruz Biotechnology; 1:10,000).

##### Cell Proliferation

LNCaP and LNCaP95 cells were pretreated for 1 h with SINT1, EPI-002, bicalutamide, enzalutamide, or vehicle before addition of 0.1 nm R1881 (LNCaP) under serum-free and phenol red-free conditions and then incubated for another 48 (LNCaP95) or 72 h (LNCaP). Cells were pulse-labeled with 10 μm BrdU for 2 h. Cells were fixed, and BrdU-labeled cells were identified with anti-BrdU-peroxidase (Roche Applied Science). BrdU incorporation was measured at 570 nm using a VersaMax ELISA microplate reader (Molecular Devices). At least three independent experiments with six technical replicates were analyzed, and average values are indicated.

##### AR Nuclear Translocation

LNCaP cells seeded on coverslips were transiently transfected with an expression vector for AR-YFP for 24 h prior to pretreatment for 1 h with 10 μm SINT1, 10 μm bicalutamide, 10 μm enzalutamide, or vehicle. Cells were exposed to 1 nm R1881 for an additional 2 h, fixed with paraformaldehyde, and mounted in Vectashield antifade mounting medium with DAPI (H-1200, Vector Laboratories). Cellular localization of AR was examined by fluorescence microscopy using an Axiovert 200 microscope equipped with a YFP filter (excitation, 500/20 nm; emission, 535/30 nm; Zeiss, Toronto, Ontario, Canada). Images were taken using a Axiocam MR camera and the AxioVision 4.4 software (Zeiss, Toronto, ON, Canada).

##### AR-FL- and AR-V7-regulated Gene Expression

LNCaP95 cells plated in 10% dextran-coated charcoal-stripped FBS, RMPI 1640 medium were pretreated with 10 μm bicalutamide, 10 μm SINT1, or 5 μm enzalutamide under serum-free and phenol-free RPMI 1640 medium conditions for 1 h before adding 1 nm synthetic androgen R1881 or vehicle (EtOH). At 48 h, total RNA was isolated using a PureLink RNA Mini kit (Invitrogen). For *in vivo* expression, tumors were harvested 3 days after last treatment, and RNA was extracted using TRIzol. Prior to cDNA generation, 4 μg of RNA were DNase-treated using DNase I (amplification grade; Sigma-Aldrich). DNase-treated RNA was split into two tubes (+RT and −RT), and cDNA was generated using the High Capacity RNA-cDNA kit (Applied Biosystems). Once complete, both reactions were adjusted to 5 ng/μl and stored at −20 °C. Approximately 5 ng of diluted cDNA and gene-specific primers were mixed with Platinum SYBR Green qPCR SuperMix-UDG with ROX (Invitrogen). The transcripts were measured using an ABI PRISM 7900 Sequence Detection System (Invitrogen). For all quantitative RT-PCR experiments, each sample was tested in triplicate, and gene expression levels were normalized to the reference gene *RPL13A*. Neuron-specific enolase (ENO2) (Gene ID, 2026; pair 1) and synaptophysin (SYP) (Gene ID, 6855; pair 2) human primers were from Sigma Life Science. All other primers were described previously (4, 9) and purchased from Integrated DNA Technologies.

##### Pharmacokinetics

Pharmacokinetics assays were performed by NAEJA Pharmaceutical Inc. in a total of 27 CD-1 mice, which received a single intravenous SINT1 dose of 50 mg/kg of body weight. Blood samples were collected during a period of 2.5 min to 8 h postdosing from three mice per time point. Plasma concentrations of SINT1 and pharmacokinetics parameters were determined.

##### Xenografts

Male NOD-SCID mice bearing subcutaneous LNCaP or LNCaP95 tumors were castrated when the tumor volume reached ∼100 mm^3^. Seven days after castration, animals were injected intratumorally with 30 mg/kg of body weight SINT1 or a matching volume of vehicle (DMSO) every 3 days for a total of 15 days. Animal behavior and body weight were observed throughout the study period for signs of toxicity. Tumor volumes were calculated by the formula length × width × height × 0.5236. Tumors were excised 3 days after the last injection and prepared for immunohistochemistry or gene expression.

##### Immunohistochemistry

Xenograft sections were stained with hematoxylin and eosin and for Ki67 expression by Wax it Histology Inc. Cells that were positive for Ki67 were counted in sections from three xenografts for each treatment. For PSA and AR staining, dewaxed sections were microwaved for 10 min on full power in prewarmed Antigen Unmasking Solution (pH 6; Vector Laboratories, Inc.). The endogenous peroxidase activity was blocked by incubation in 3% H_2_O_2_ for 10 min followed by incubation in Clear Vision Histostaining Blocking Solution (UniCure Lab, Inc.) for 1 h at room temperature. Sections were incubated with AR-N20 (1:500 dilution) and PSA (1:5 dilution; overnight at 4 °C) primary antibodies. Samples were then incubated for 30 min at room temperature with secondary antibodies and mounted in Permount mounting medium (SP15–100, Fisher).

##### Statistics

One-way ANOVA Dunnett's multiple comparison test was performed using GraphPad software unless stated otherwise, and differences were considered statistically significant at *p* values less than 0.05.

## Results

### 

#### 

##### SINT1 Specifically Inhibits AR Transcriptional Activity

**FIGURE 1.**
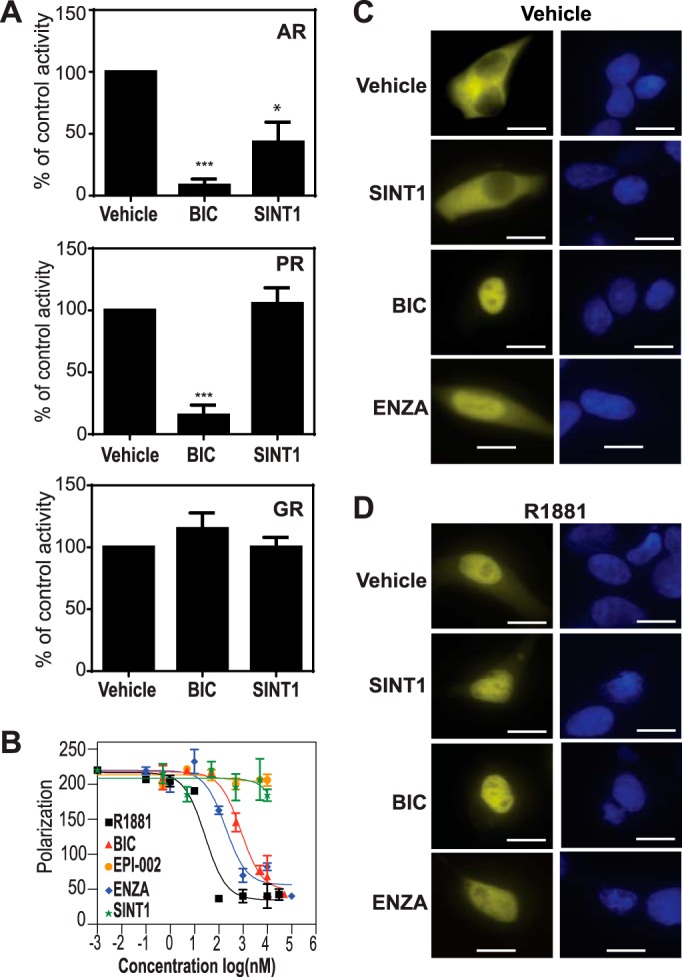
**SINT1 specifically inhibits AR transcriptional activity.** *A*, LNCaP cells were transfected with PSA (6.1 kb)-luciferase, PRE-luciferase, or GRE-luciferase reporters and expression vector for PRβ or GRα. Cells were pretreated with 10 μm SINT1, 10 μm bicalutamide, or vehicle before exposure to the corresponding ligand (1 nm R1881, 10 nm 4-pregnene-3,20-dione, or 10 nm dexamethasone). *Error bars* represent mean percentage of vehicle activity ±S.E. of at least three independent experiments with triplicate wells. SINT1, 10 μm; bicalutamide (*BIC*), 10 μm. *, *p* < 0.05; ***, *p* < 0.001. *B*, representative competition binding curve showing displacement of fluorescently labeled ligand (Fluoromone) from recombinant AR LBD by increasing concentrations of bicalutamide, EPI-002, enzalutamide (*ENZA*), SINT1, or agonist R1881. *Error bars* represents mean ± S.E. of three technical replicates. *C*, in the absence of androgen, nuclear translocation of YFP-AR in LNCaP cells treated for 1 h with SINT1, bicalutamide, enzalutamide, or vehicle (DMSO). *D*, in the presence of androgen (R1881 at 1 nm), nuclear translocation of YFP-AR in LNCaP cells treated with SINT1, bicalutamide, enzalutamide, or vehicle. DAPI staining indicates the location of the nucleus. *Scale bars*, 20 μm.

AR has high sequence homology with related steroid hormone receptors such as PR and GR in their DBDs and LBDs. These related steroid hormone receptors also interact with many of the same coactivators and other proteins. Therefore, to determine the specificity of SINT1 for AR, we tested whether SINT1 would inhibit PR or GR transcriptional activities. SINT1 significantly inhibited androgen-induced activity of endogenous AR using the synthetic androgen R1881 (Fig. 1*A*), which was consistent with a previous report (7). Importantly, SINT1 did not affect the transcriptional activities of PR or GR (Fig. 1*A*). The antiandrogen bicalutamide inhibited both AR and PR transcriptional activities as reported previously (10). These data support that SINT1 specifically inhibits AR transcriptional activity and that it is not a general inhibitor of transcription or translation because neither GR nor PR transcriptional activities were decreased.

##### SINT1 Does Not Bind AR LBD

To determine whether the mechanism of action of SINT1 to inhibit androgen-induced AR transcriptional activity was mediated by binding to AR LBD, a fluorescence polarization competition assay was used. This assay uses recombinant AR LBD to reveal whether a test compound competes for ligand binding of Fluoromone that binds to this domain. R1881, bicalutamide, and enzalutamide are all known ligands of AR LBD, and as expected each competed with Fluoromone (Fig. 1*B*). SINT1 and EPI-002 (negative control) (4) did not compete with Fluoromone for binding to AR LBD in the concentration range of 0.5–50 μm. These data revealed that SINT1 does not interfere with ligand binding to AR LBD and suggests that SINT1 is not a ligand of AR LBD.

##### SINT1 Does Not Affect Nuclear Localization of AR

AR signaling involves its nuclear translocation upon binding of ligand to the LBD. Antiandrogens are LBD ligands that can induce nuclear translocation of AR in the absence of androgen. In the absence of androgen, AR remained cytoplasmic in cells treated with SINT1 (Fig. 1*C*) contrary to antiandrogens that induced nuclear translocation as reported (5, 11–13). Thus, SINT1 behaves differently than ligands of AR LBD. Next we tested whether SINT1-decreased transcriptional activity of AR in response to androgen was mediated by a mechanism of preventing nuclear translocation of AR. Androgen induced AR translocation to the nucleus regardless of treatments with SINT1 or antiandrogens (Fig. 1*D*). These data suggest that the mechanism of action of SINT1 to decrease androgen-induced AR transcriptional activity was not by preventing nuclear translocation of AR. The fact that SINT1 does not bind to the LBD is consistent with it not inducing nuclear translocation in the absence of androgens like known LBD ligands that include androgens and antiandrogens.

##### SINT1 Binds Endogenous FL-AR in Living Cells

**FIGURE 2.**
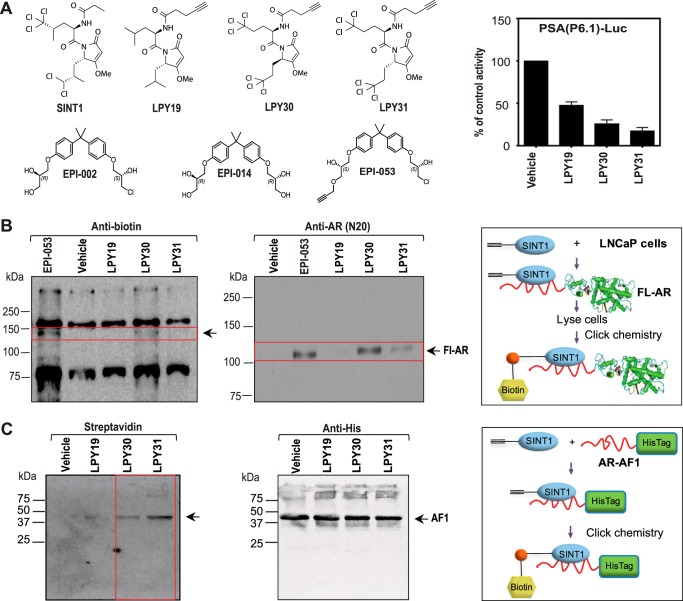
**SINT1 binds endogenous AR in living cells to inhibit transactivation of AR NTD.** *A*, chemical structures of EPI-002, EPI-053, LPY19, LPY30, LPY31, and SINT1 (*left*). The effect of SINT1 probes in AR transcriptional activity was tested in LNCaP cells transiently transfected with PSA (6.1 kb)-luciferase and exposed for 1 h to 40 μm LPY19 and 20 μm LPY30 and LPY31 before induction with 1 nm R881 for 48 h. *Error bars* represent the mean ± S.E. of *n* = 3 independent experiments with triplicate wells (*right*). *B*, binding of SINT1 click chemistry analogs to FL-AR in cells. LNCaP cells were exposed to modified SINT1 (LPY19, -30, and -31), EPI-053, or DMSO vehicle overnight prior to preparing whole cell lysates for click chemistry reactions. Biotinylated SINT1 or EPI probes bound to proteins were captured with streptavidin-agarose resin prior to separation by 10% SDS-PAGE and subjected to Western blotting analysis with antibodies directed to either biotin (*left*) or AR (*middle*). *Red boxes* highlight where AR migrates on the gel. *Right*, schema of experiment. *C*, purified recombinant AF1-His tag protein was incubated with LPY19 (inactive), LPY30 and LPY31 (both active), or vehicle (DMSO) prior to click chemistry for biotin labeling, SDS-PAGE, and detection of biotin-labeled probes covalently bound to AF1-His tag (*left*). Western blotting analysis of the same membrane using an antibody to His tag revealed equal loading of AF1-His tag protein in each lane (*middle*). *Right*, schema of experiment.

To reveal whether SINT1 binds to endogenous AR, LNCaP cells were incubated with modified SINT1 click chemistry probes (LPY19, LPY30, and LPY31 shown in Fig. 2*A*). The EPI-002 click chemistry probe EPI-053 was used as a positive control (5). LPY30, LPY31, and EPI-053 all had biological activity as measured using an AR-driven reporter gene construct (Fig. 2*A*, *right*, and Ref. 5). LPY19 had very weak activity with an IC_50_ of ∼40 μm and was considered inactive at 20 μm. LNCaP cells were incubated overnight with these probes prior to click chemistry to add biotin to each click chemistry probe (see schema in Fig. 2*B*, *right*). Cell lysates were used for immunoprecipitation with streptavidin to isolate those proteins bound to biotinylated probes. Immunocomplexes were split with one aliquot used for Western blotting analysis using an antibody to biotin (Fig. 2*B*, *left*) and the other aliquot for Western blotting analysis using an antibody to AR (Fig. 2*B*, *middle*). Antibodies against biotin revealed a band corresponding to AR only with cells treated with active analogs EPI-053, LPY30, and LPY31 (very faint band) but not in those exposed to inactive analog LPY19 or the DMSO vehicle control (Fig. 2*B*, *left*). Consistent with these biotin bands corresponding to AR, the AR was immunoprecipitated with streptavidin only in those immunocomplexes that were both positive for biotin (Fig. 2*B*) and biologically active against AR transcriptional activity (EPI-053, LPY30, and LPY31).

##### SINT1 Binds AF-1

SINT1 binds to endogenous FL-AR (Fig. 2*B*) but does not affect ligand binding to AR LBD (Fig. 1*B*). Therefore, we next evaluated whether SINT1 binds the AF-1 region in AR NTD. Purified recombinant AF-1 protein (AF1-His tag) was incubated under cell-free conditions with LPY19, LPY30, and LPY31 prior to click chemistry to add a biotin to the LPY probe (see schema in Fig. 2*C*, *right*). Western blotting analysis using streptavidin revealed binding of the biotinylated LPY30 and LPY31 probes to AF-1 but no binding of the inactive negative control LPY19 (Fig. 2*C*, *left*). Using an antibody to His tag revealed equal loading of AF-1 protein to each lane (Fig. 2*C*, *middle*). These data show that SINT1 click chemistry probes that inhibit AR transcriptional activity bind to the AR AF-1 region, whereas inactive probe does not bind AF-1.

##### SINT1 and EPI-002 Potentially Bind Different Regions of AF-1

To test whether SINT1 can inhibit transactivation of AR NTD, LNCaP cells were co-transfected with an expression vector for AR NTD encoding amino acids 1–558 fused to the Gal4 DBD and a reporter construct with Gal4 DBD-binding sites. Transactivation of AR NTD is induced by forskolin or IL-6 (8, 14).

**FIGURE 3.**
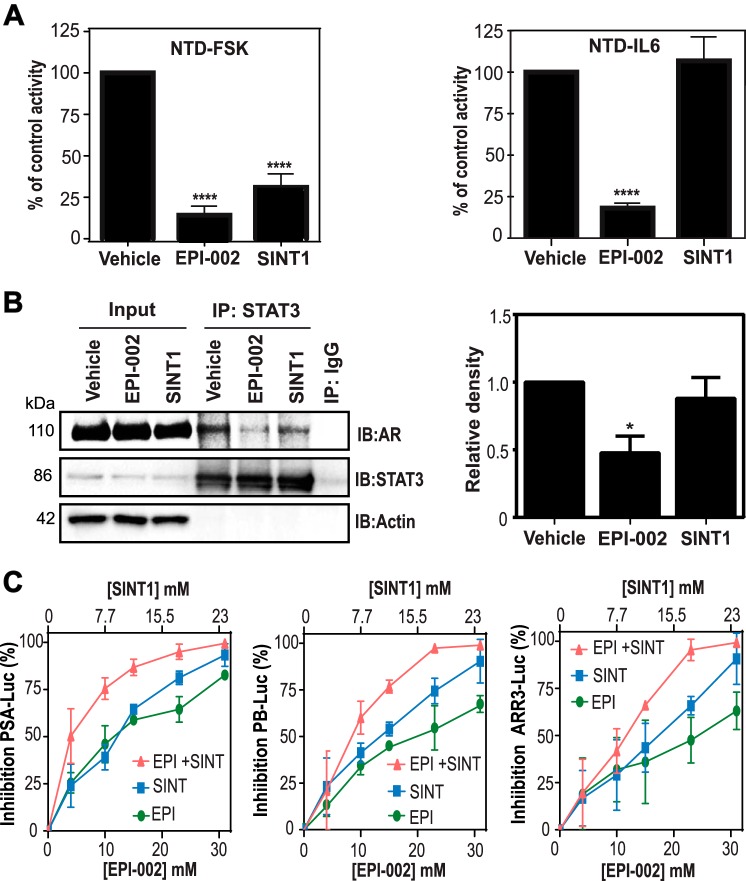
**EPI-002 and SINT1 potentially target different regions of AR AF-1.** *A*, transactivation assay of AR NTD in LNCaP cells co-transfected with Gal4UAS-TATA-luciferase and AR(1–558)-Gal4 DBD treated for 1 h with EPI-002, SINT1, or DMSO vehicle control followed by 24 h of induction of transactivation by incubation with forskolin (*FSK*) (*left*) or IL-6 (*right*). Luciferase activities were normalized to protein concentration and are presented as percentage of vehicle control. *Error bars* represent the mean ± S.E. of *n* = 4 independent experiments with triplicate wells. One-way ANOVA Dunnett's multiple comparison test was used for statistical analyses. ****, *p* < 0.0001. *B*, LNCaP cells were serum-starved and exposed to IL-6 for 6 h. Whole cell lysates were precleared with mouse IgG, immunoprecipitated with anti-STAT3 antibodies (*IP*), and then analyzed by immunoblotting (*IB*). Relative densities of AR in immunoprecipitated samples were normalized to STAT3 levels. *Error bars* are mean ± S.E. of *n* = 3 independent experiments with triplicate wells. One-way ANOVA Bonferroni's multiple comparison test was used for statistical analyses comparing the treatment groups with each other. *, *p* < 0.05. *C*, combination treatments in LNCaP cells co-transfected with PSA-Luc, PB-Luc, and ARR3-Luc. Cells were treated with SINT1, EPI-002, or a combination at a 1:1.3 ratio followed by R1881 treatment. *Error bars* represent the mean percentage inhibition ±S.E. of *n* = 3 independent experiments.

Forskolin-induced transactivation of AR NTD was significantly inhibited by SINT1 and EPI-002 (positive control) (Fig. 3*A*, *left*). Surprisingly, SINT1 had no significant effect in blocking transactivation of AR NTD induced by IL-6, whereas EPI-002 was a potent inhibitor (Fig. 3*A*, *right*) as reported (4). STAT3 interacts with the AR NTD in response to IL-6 to act as a coactivator of AR (14). Consistent with EPI blocking IL-6-induced transactivation of the AR NTD, EPI inhibited interaction between AR and STAT3, whereas SINT1 did not (Fig. 3*B*). Together these data suggest that SINT1, similarly to EPI, binds the AR AF-1 region, but SINT1 may have a different binding site on AF-1 compared with EPI compounds. This is because, unlike EPI, SINT1 had no effect on IL-6 transactivation of AR NTD and did not block STAT3 interaction with AR; however, it significantly inhibited forskolin-induced transactivation of AR NTD.

##### Combinations of EPI with SINT1 Suggest Different Mechanisms of Action

A standard approach to determine different mechanisms of two compounds is to examine whether combination treatment results in additive or synergistic responses compared with each monotherapy. Here we undertook these studies using three AR-driven reporters with SINT1 and EPI-002 used alone or in combination. The IC_50_ values for SINT1 were 10.74 ± 1.55, 11.64 ± 0.78, and 13.22 ± 1.19 μm for PSA (6.1 kb)-Luc, PB-Luc, and ARR3-Luc, respectively. When SINT1 was combined with EPI-002, the curve shifted left with these two compounds having an additive effect on all three reporters at all concentrations except the lowest dose (Fig. 3*C*). The median combination index for PSA (6.1 kb)-Luc, PB-Luc, and ARR3-Luc reporter were 1.001, 1.016, and 1.012, confirming an additive effect. These data support that SINT1 and EPI-002 may have different binding sites on AR AF-1 and concomitantly different mechanisms of action.

##### SINT1 Inhibits Constitutively Active AR-V567es Splice Variant

**FIGURE 4.**
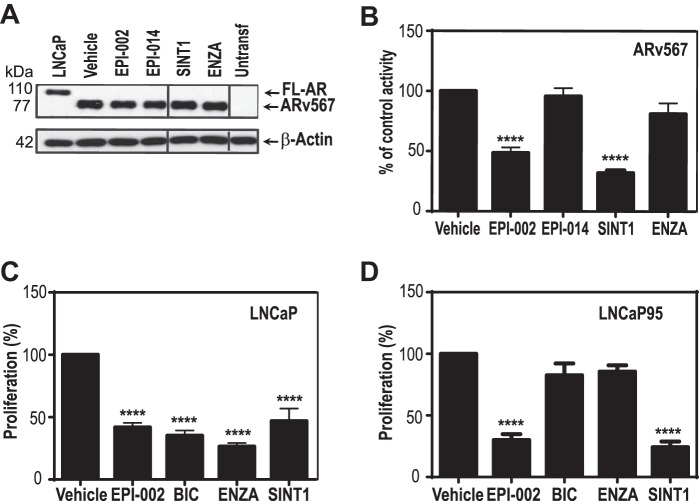
**SINT1 inhibits AR splice variant and FL-AR transcriptional activities.** The effect of SINT1 on AR splice variant AR-v567es is shown. *A*, whole cell protein lysates from cells treated in *B* were analyzed by Western blotting to reveal levels of forced expression of AR-V567es compared with endogenous FL-AR in LNCaP cells. *B*, COS-1 cells co-transfected with PB-Luc and an expression vector for AR-V567es prior to treatment with EPI-002, EPI-014 (inactive), SINT1, enzalutamide (*ENZA*), or DMSO vehicle control. Luciferase activities were normalized to protein concentrations of the samples. *Error bars* represent the mean ± S.E. of *n* = 3 separate experiments with triplicate wells. The effect of SINT1 on proliferation of cells expressing FL-AR (*C*) and constitutively active AR splice variant (*D*) is shown. LNCaP cells treated with 0.1 nm R1881 (*C*) and LNCaP95 cells (*D*) were exposed to EPI-002, bicalutamide (*BIC*), enzalutamide, SINT1, or DMSO vehicle control for 72 or 48 h, respectively. *Error bars* represent the mean percentage of vehicle control ±S.E. of *n* = 3 independent experiments with six technical replicates. One-way ANOVA Dunnett's multiple comparison test was used for statistical analyses. ****, *p* < 0.0001.

Expression of AR splice variants such as AR-V7 or AR-V567es is a proposed mechanism of resistance to current hormone therapies for prostate cancer (15–18). An antagonist of AR AF-1 could potentially overcome the failure of current therapies directed to AR LBD with EPI providing proof of principle of a therapeutic response for CRPC (4, 5). Given that, similarly to EPI, SINT1 blocks transactivation of AR NTD and binds to AF-1, SINT1 could antagonize the activity of constitutively active splice AR variants that lack the LBD. Therefore, the ability of SINT1 to inhibit the transcriptional activity of AR-V567es was tested. Levels of AR-V567es protein ectopically expressed in COS-1 cells were within the physiological range of endogenous FL-AR in LNCaP cells, and these levels were not decreased by treatments with SINT1 (Fig. 4*A*). Under these conditions, AR-V567es transcriptional activities were significantly inhibited by SINT1 (Fig. 4*B*) and EPI-002 (positive control) but not by the inactive analog EPI-014 (also known as 185-9-1 or bisphenol A bis(2,3-dihydroxypropyl) ether (BADGE·2H_2_O)) (4).

Previously SINT1 was shown to block androgen-dependent growth of LNCaP cells but to have no effect on PC3 cells that do not depend on AR for growth and survival (7). Consistent with those data, androgen-induced proliferation of LNCaP cells that express FL-AR was inhibited by antiandrogens that target AR LBD as well as EPI-002 and SINT1, which both block activity of AR AF-1 (Fig. 4*C*). Importantly, an AF-1 antagonist should block transcriptional activity of AR splice variants that lack LBD. Therefore, next we determined whether SINT1 could block endogenously expressed AR variants using LNCaP95 cells. These are androgen-independent cells that express both FL-AR and constitutively active AR splice variants that lack LBD (19, 20). Proliferation of LNCaP95 cells is driven by constitutively active AR-V7 (20). LNCaP95 cells were treated with SINT1, EPI-002, enzalutamide, and bicalutamide. Proliferation of LNCaP95 cells was inhibited by SINT1 and EPI-002, whereas there was no effect with antiandrogens (Fig. 4*D*), consistent with other reports that these cells are resistant to antiandrogens including enzalutamide (21, 22). Together these results support that SINT1 targets AR AF-1 to inhibit the transcriptional activities of constitutively active AR splice variant and FL-AR.

##### SINT1 Does Not Affect Levels of FL-AR or AR-V7 Expression but Does Inhibit Expression of Their Target Genes

**FIGURE 5.**
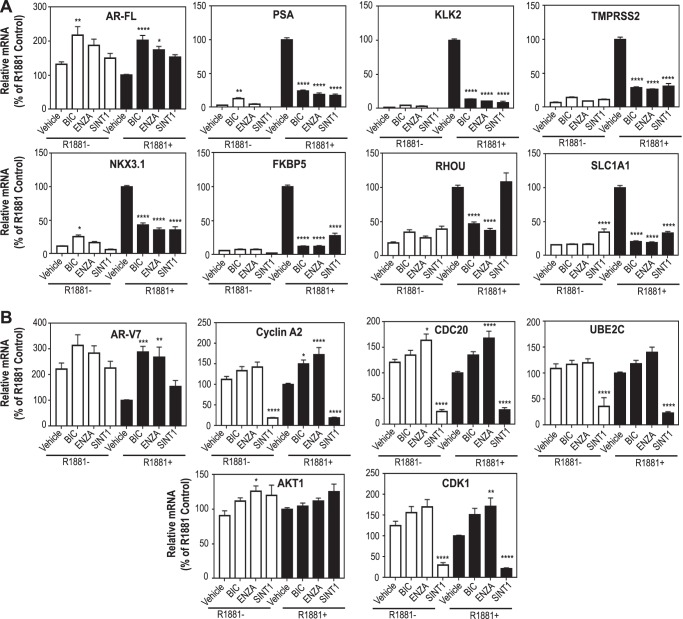
**SINT1 decreases levels of expression of both FL-AR- and AR-V7-regulated genes.** LNCaP95 cells were treated with vehicle (DMSO), bicalutamide (10 μm), enzalutamide (5 μm) or SINT1 (10 μm) prior addition of R1881 for 48 h. *A*, Transcript levels of FL-AR and its regulated genes *KLK3*/*PSA*, *KLK2*, *TMPRSS2*, *NKX3.1*, *FKBP5*, *RHUO*, and *SLC1A1. B*, transcript levels of AR-V7 and its regulated genes cyclin A2, *CDC20*, *UBE2C*, *AKT1*, and *CDK1*. Levels of transcripts were normalized to *RPL13*, a housekeeping gene. Data are presented as percentage of controls in the presence of androgen. *Error bars* represent mean ± S.E. of *n* = 4 independent experiments. *, *p* < 0.05; **, *p* < 0.01; ***, *p* < 0.001; ****, *p* < 0.0001. One-way ANOVA Tukey's multiple comparison test was used for statistical analyses.

The ability of SINT1 to repress the expression of endogenous genes regulated by both FL-AR and AR-V7 was also evaluated using LNCaP95 cells. SINT1 did not lead to an increase in levels of FL-AR mRNA when compared with its relevant vehicle control, contrary to antiandrogens (Fig. 5*A*). Androgen-induced levels of transcripts of FL-AR-regulated genes (*KLK3*/*PSA*, *KLK2*, *TMPRSS2*, *NKX3.1*, *FKBP5*, and *SLC1A1*) were all significantly repressed by SINT1 with the exception of *RHOU*. These data support that SINT1 inhibits FL-AR transcriptional activity and that SINT1 blockade may be gene-specific, which may be due to its unique mechanism of action compared with antiandrogens.

SINT1 also had no significant effect on levels of AR-V7 transcript unlike bicalutamide and enzalutamide (Fig. 5*B*). Evidence to support that SINT1 inhibits the transcriptional activity of AR splice variants, as shown in Fig. 4, *B* and *C*, was provided by measurement of levels of endogenous expression of AR-V7 target genes. SINT1 significantly repressed levels of cyclin A2, CDC20, UBE2C, and CDK1 transcripts. AKT1 transcript levels were not repressed by any treatment. SINT1 blocks the expression of target genes regulated by FL-AR and AR splice variants, which is consistent with what would be expected from an inhibitor that binds to AR AF-1.

##### SINT1 Causes Regression of CRPC Tumors

SINT1 is labile, and over the course of completing these studies its activity decreased. Structural examination revealed a potential to be quickly metabolized in biological systems. Therefore, we first evaluated its pharmacokinetics before *in vivo* studies were carried out to determine dose and route of delivery. The maximum concentration (*C*_max_) in plasma following a single intravenous dose of 50 mg/kg of body weight was 4.15 μg/ml (∼8 μm), which was slightly lower than its IC_50_ *in vitro*. SINT1 had a short elimination half-life (*t*_½_) of 1.16 h, the volume of distribution was 36.59 liter/kg, and the plasma clearance was 21.93 liter/h/kg (Table 1).

**TABLE 1:** **Pharmacokinetic parameters of SINT1** AUC_inf_, area under the concentration curve; *C*_max_, maximum concentration; Cl, clearance; *t*½, plasma half-life; *V_d_*, volume of distribution.

Mice/Set	Parameter				
	AUC_inf_	*C*_max_	Cl	*t*½	*V_d_*
	*h* × μ*g*/*ml*	μ*g*/*ml*	*liter*/*h*/*kg*	*h*	*liter*/*kg*
1	1.84	3.99	21.96	1.38	43.66
2	2.37	4.44	17.04	1.10	27.14s
3	1.51	4.03	26.80	1.01	38.96
Mean ± S.D.	1.90 ± 0.43	4.15 ± 0.25	21.93 ± 4.88	1.16 ± 0.19	36.59 ± 8.51

**FIGURE 6.**
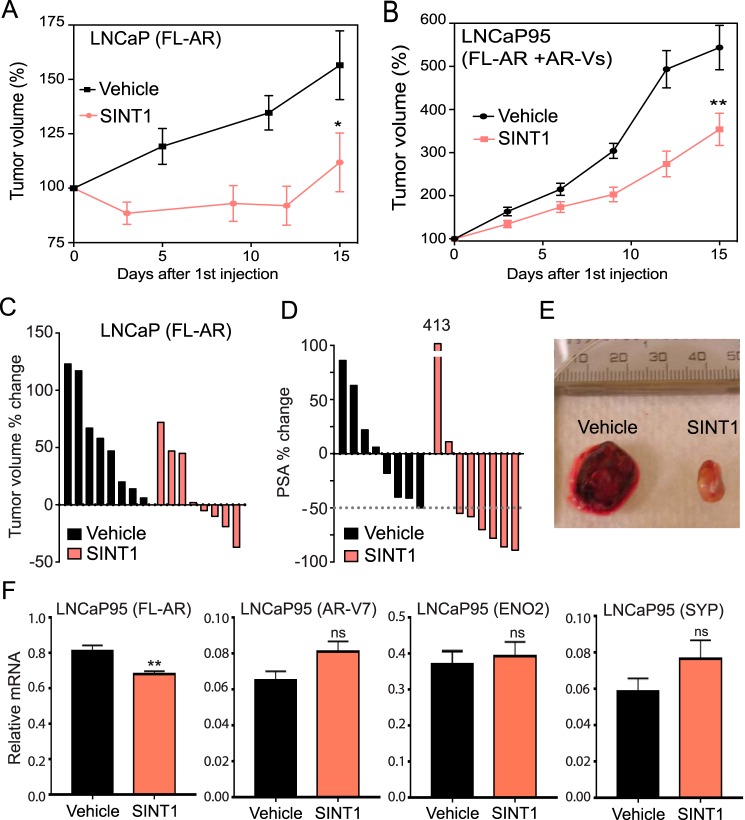
***In vivo*, SINT1 inhibits the growth of CRPC expressing solely FL-AR or both FL-AR and AR splice variants.** Tumor growth (*A*, LNCaP cells expressing FL-AR; *B*, LNCaP95 expressing FL-AR and AR splice variants) in castrated mice that received intratumoral doses of SINT1 (30 mg/kg of body weight) for a total of five doses or matching vehicle (DMSO) every 3 days is shown. *Error bars* represent the mean ± S.E. of *n* = 8. *C*, individual animal tumor volume change of the LNCaP tumors from *A* at the duration of the experiment on day 15. *D*, day 15 serum PSA percent change from the day of first dose. The *dotted gray line* indicates a 50% drop in serum PSA levels. *E*, photograph of harvested representative LNCaP tumors from animals that received SINT1 or vehicle (DMSO) treatment. *F*, effect of SINT1 on expression levels of AR-FL, AR-V7, ENO2, and SYP in LNCaP95 xenographs from *B*. Unpaired *t* test was used for statistical analyses. *Error bars* represent the mean ± S.E. of *n* = 9. *, *p* < 0.05; **, *p* < 0.01; *ns*, not significant.

*In vitro* data support that SINT1 might have antitumor effects on CRPC tumors by targeting AF-1. Thus, inhibition of tumor growth was evaluated in castrated mice bearing LNCaP xenografts receiving SINT1 by intratumoral delivery to ensure that SINT1 reached the tumors unchanged by metabolism. LNCaP xenograft volumes were ∼125 mm^3^ on the day of first dose with no significant difference in starting tumor volume between the two groups. SINT1 significantly inhibited CRPC tumor growth with very little overall growth (112 ± 13%, *n* = 8; *p* = 0.0495) compared with DMSO-treated tumors (157 ± 16%, *n* = 8) (Fig. 6*A*). SINT1 was also able to significantly reduce the growth of aggressive fast doubling LNCaP95 tumors that express both FL-AR and AR-Vs (Fig. 6*B*). Importantly, SINT1 caused tumor regression in four of seven CRPC LNCaP xenografts (Fig. 6*C*), and tumors had a less bloody appearance (Fig. 6*E*). In these animals, a serum PSA drop of ≥50% was achieved in six animals treated with SINT1 but in only one animal in the control group (Fig. 6*D*). Levels of FL-AR and AR-V7 transcripts were not significantly altered by SINT1 treatment in harvested LNCaP95 xenografts (Fig. 6*F*) nor was there support that SINT1 induced neuroendocrine differentiation as indicated by no significant changes in levels of expression of *ENO2* and *SYP* genes (Fig. 6*F*).

##### SINT1 Decreases Proliferation and Expression of PSA in CRPC Tumors

**FIGURE 7.**
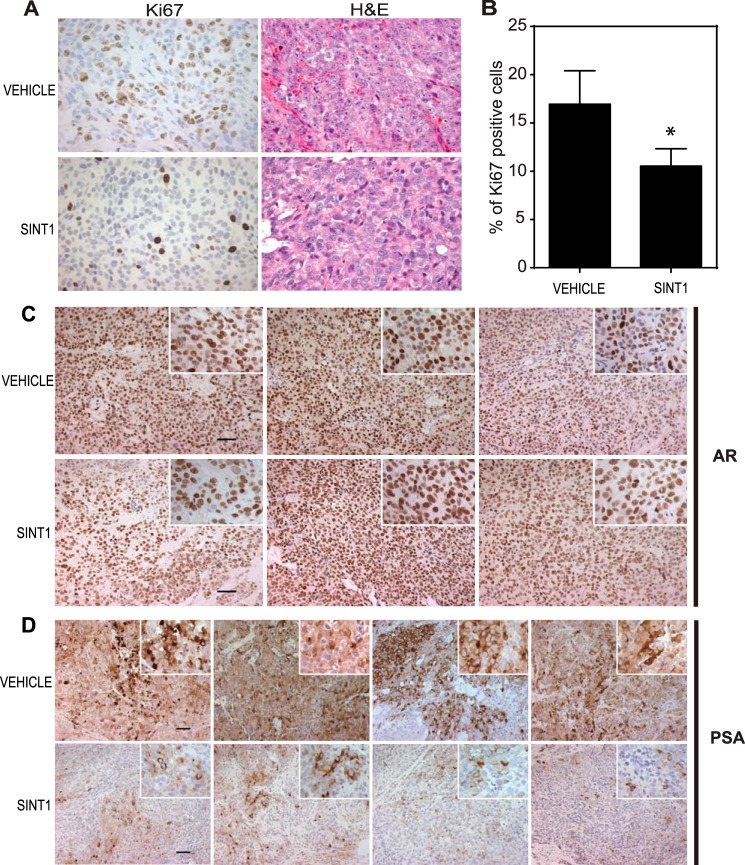
**SINT1 decreases proliferation and expression of PSA in CRPC xenografts.** *A*, immunohistochemistry of sections of LNCaP xenografts harvested at the duration of the experiment and stained for Ki67 and hematoxylin and eosin (H&E). *B*, quantification of tissue samples stained for proliferation marker Ki67. Percentages of Ki67-positive cells were counted in sections from three xenografts for each treatment. At least 1100 cells per xenograft were counted. The total numbers of cells counted were 4323 (DMSO) and 5000 (SINT1). *Error bars* represent the mean ± S.E. of *n* = 3. Unpaired *t* test was used for statistical analyses. *, *p* < 0.05. *C*, staining for AR in three representative harvested xenografts from vehicle control-treated animals or SINT1-treated animals. *D*, PSA staining of four representative xenografts from vehicle control-treated animals or SINT1-treated animals. *Scale bar*, 20 μm.

LNCaP xenografts harvested at the duration of the experiment were analyzed by immunohistochemistry staining for the proliferation marker Ki67 and for expression of AR and PSA. Consistent with SINT1 decreasing tumor volume, SINT1 also reduced the number of Ki67-positive cells in harvested xenografts (Fig. 7, *A* and *B*). Levels and cellular localization of AR protein were similar in xenografts from vehicle-treated and SINT1-treated animals (Fig. 7*C*). Importantly, there were marked decreases in the levels of PSA in xenografts treated with SINT1 compared with the vehicle control (Fig. 7*D*), which was consistent with the 50% drop in serum PSA levels in the majority of SINT1-treated animals. Together these *in vivo* data with the *in vitro* data support that SINT1 inhibits the transcriptional activities of FL-AR and AR-Vs.

## Discussion

Treatment of mCRPC with second generation inhibitors of the androgen axis such as abiraterone or enzalutamide has increased overall survival (23, 24). Unfortunately, resistance to these therapies is inevitable, and eventually patients will succumb to mCRPC (25–27). Despite initial responses to inhibitors of the androgen axis, resumed AR signaling is still considered to drive most mCRPC (for a review, see Ref. 28).

Several mechanisms of resistance that could drive AR transcriptional activity despite application of enzalutamide or abiraterone include gain-of-function mutations of AR, breakthrough of androgen synthesis, and expression of constitutively active AR splice variants (15–19, 29–32). Of these mechanisms, clinical evidence based on detection of constitutively active AR splice variant in circulating tumor cells from patients with CRPC suggests a correlation with resistance to enzalutamide and abiraterone (17, 18). Currently there are no approved therapies in the clinic that inhibit the activity of these truncated constitutively active ARs that lack the LBD. Therefore, developing drugs such as antagonists to AR AF-1 that target both truncated constitutively active AR variants and FL-AR is essential to achieve complete AR blockade and could potentially improve the clinical management of mCRPC.

Here we characterized an antagonist to AR NTD, SINT1, that may be able to overcome resistance linked to expression of AR splice variants or other AR-dependent mechanisms of resistance. Specifically we showed that SINT1 1) directly binds AF-1, 2) inhibited forskolin-induced transactivation of AR NTD, 3) attenuated transcriptional activities of both FL-AR and AR splice variant, 4) decreased FL-AR- and AR-V7-dependent proliferation of prostate cancer cells, and 5) *in vivo* decreased the growth of CRPC tumors concomitant with decreased serum levels and expression of *PSA*, a clinically relevant AR-regulated gene. Changes in serum PSA are the earliest indication of tumor response and precede any changes in clinical symptoms (33). Specificity of SINT1 for AR was previously suggested based upon data that SINT1 blocked AR-dependent proliferation but had no effect on cells that do not depend on AR for growth or survival (7). Data presented here support these conclusions and were provided by multiple approaches. First, Western blotting analyses of immunocomplexes of biotinylated SINT1 from LNCaP cells treated overnight with click chemistry probes (Fig. 2*B*) show a profound lack of protein bands detected by an antibody to biotin with the exception of AR. Second, SINT1 inhibited AR transcriptional activity but had no inhibitory effect on structurally related proteins PR and GR. AR DBD and LBD have ∼80 and 50% sequence homology, respectively, with PR and GR. AR NTD has less than 15% sequence homology with steroid hormone receptors, thereby making it an attractive drug target. However, the small regions of AR NTD that are conserved with other steroid hormone receptors are amino acid residues 1–30, 153–169 (47% with PR), and 236–247 (75% with GR). These regions are important for androgen-dependent transcriptional activity. Region 1–30 contains core residue sequence ^23^FQNLF^27^ required for optimal orientation and interaction of AR NTD with AR LBD for AR antiparallel dimer formation in response to ligand (34). The conserved residues 153–169 and 236–247 are within Tau1 (residues 101–370), but neither of these regions include the core sequence ^178^LKDIL^182^. Tau1 is suggested to be required for transcriptional activity of FL-AR in response to androgen. Seminal work by Brinkmann and co-workers (3) showed that deletion of residues 1–359 of a truncated constitutively active AR lacking the LBD did not decrease its transactivation capacity. These data suggest that Bet bromodomain inhibitors such as JQ1 that block interaction of Brd4 with residues 120–160 of the AR NTD (35) may not have any effect on constitutively active AR splice variants because that region of the NTD is not critical for androgen-independent activity. Tau5 (residues 360–485) with core sequence ^435^WHTLF^439^ appears to be vitally important for AR transcriptional activity in the absence of androgen and possibly also for constitutively active truncated AR splice variants. NMR spectroscopy show that EPI-001 binds in a pocket in Tau5 with contact to three regions, residues 353–364, 397–407, and 433–466 (36). These data are consistent with biological data showing that EPI analogs inhibit the transcriptional activities of androgen-dependent and -independent FL-AR and constitutively active truncated ARs lacking the LBD including AR splice variants (4, 5).

**FIGURE 8.**
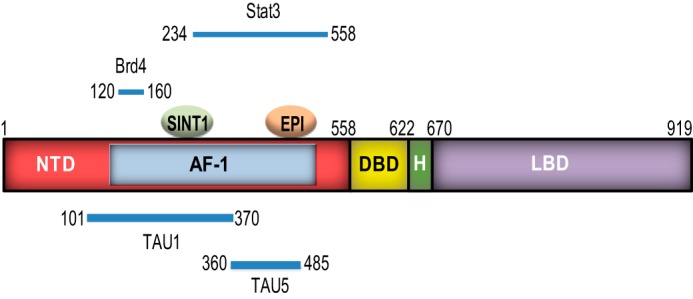
**Proposed binding model of SINT1.** EPI binds Tau5 (36), whereas SINT1 may bind more toward the N terminus, perhaps overlapping into Tau1 (Figs. 2 and 3). STAT3 binds within the region encoding residues 234–558 (14). Brd4 binds within residues 120–160 (35). *H*, hinge region.

Here the ability of SINT1 to inhibit constitutively active AR splice variant was shown directly in a transcriptional assay with AR-V567es and an AR-driven reporter gene construct as well as indirectly by blocking the growth of LNCaP95 cells that are reported to be driven by AR-V7 (20). Endogenous expression of genes regulated by FL-AR and AR-Vs was attenuated by SINT1, consistent with it blocking transcriptional activities of FL-AR and AR-Vs. The inhibitory effect on AR transcriptional activity by SINT1 was additive with EPI-002, a Tau5 inhibitor. Unlike EPI-002, the inability of SINT1 to inhibit IL-6-induced transactivation of AR NTD or block the physical interaction between endogenous AR and STAT3 suggests the possible existence of multiple hot spots in AF-1 and that EPI and SINT1 are not in competition for a common binding site. IL-6 increases transactivation of AR NTD by direct interaction of STAT3 with a region within residues 234–558 of AR NTD (14). Because SINT1 binds to AF-1 recombinant protein that encodes residues 142–485 and is additive to EPI that binds Tau5, we hypothesize that SINT1 may bind more toward the N terminus of AF-1, perhaps overlapping or within Tau1. Fig. 8 is a model proposing potential binding sites for SINT1 and EPI based on the current knowledge. Together these data imply that multiple binding sites within the AF-1 region of AR exist and can be targeted independently. Drawing on drug development to c-Myc, at least three binding sites in its intrinsically disordered regions have been identified that can act as binding sites for seven small molecules independently and simultaneously (37). In the event that the binding site of SINT1 overlaps with Tau1 as suggested here, combination treatment with a Tau5 inhibitor such as EPI-002 could provide a mixture that would prevent a possible resistance mechanism of switching between Tau1 and Tau5. These studies are currently under intense investigation.

## Author Contributions

C. A. B. performed and analyzed the experiments shown in Figs. 1*C* and 4, *B* and *C*, and contributed to the preparation of the figures and manuscript. I. T. performed and analyzed the experiments shown in Figs. 1*A* and 4*A*. A. H. T. performed and analyzed the experiments shown in Fig. 2*C*. D. P. C. did the quantitative RT-PCR work in Figs. 5 and 6*F*. N. R. M. performed and analyzed the experiments shown in Figs. 2, *A* (*right*), and *B*, and 3*A*. Z. L. performed histochemistry staining shown in Fig. 6*C*. J. W. performed the animal experiments in Fig. 6. Y. C. Y. performed and analyzed the experiments shown in Fig. 1*B*. Y. I. performed and analyzed the experiments shown in Fig. 3*B*. L. Y. synthesized YLP19, -30 and -31. J. G. W. critically reviewed the intellectual content of the paper. R. J. A. extracted and purified SINT1 and synthesized YLP19, -30, and -31 as well as EPI-053. M. D. S. conceived and coordinated the study, wrote the paper, analyzed results, and designed Fig. 7. All authors reviewed the results and approved the final version of the manuscript.

## Footnotes

2 The abbreviations used are:

LBD: ligand-binding domain
DBD: DNA-binding domain
AR: androgen receptor
CRPC: castration-resistant prostate cancer
mCRPC: metastatic castration-resistant prostate cancer
PSA: prostate-specific antigen
AF-1: activation function-1
NTD: N-terminal domain
FL-AR: full-length AR
Tau: transcriptional activation unit
SINT1: sintokamide A
PR: progesterone receptor
GR: glucocorticoid receptor
PB: probasin
GRE: glucocorticoid response element
PRE: progesterone response element
Luc: luciferase
ANOVA: analysis of variance
AR-V: AR splice variant
ENO2: neuron-specific enolase
SYP: synaptophysin.
